# No ambiguity: Chemosensory-based ayurvedic classification of medicinal plants can be fingerprinted using E-tongue coupled with multivariate statistical analysis

**DOI:** 10.3389/fphar.2022.1025591

**Published:** 2022-12-02

**Authors:** Rama Jayasundar, Somenath Ghatak, Dushyant Kumar, Aruna Singh, Preeti Bhosle

**Affiliations:** Department of NMR, All India Institute of Medical Sciences, New Delhi, India

**Keywords:** chemosensory, ayurvedic pharmacology, *rasa*, medicinal plants, E-tongue, ayurveda, taste

## Abstract

**Background:** Ayurveda, the indigenous medical system of India, has chemosensory property (*rasa*) as one of its major pharmacological metric. Medicinal plants have been classified in Ayurveda under six *rasas*/tastes—sweet, sour, saline, pungent, bitter and astringent. This study has explored for the first time, the use of Electronic tongue for studies of *rasa*-based classification of medicinal plants.

**Methods:** Seventy-eight medicinal plants, belonging to five taste categories (sweet, sour, pungent, bitter, astringent) were studied along with the reference taste standards (citric acid, hydrochloric acid, caffeine, quinine, L-alanine, glycine, β-glucose, sucrose, D-galactose, cellobiose, arabinose, maltose, mannose, lactose, xylose). The studies were carried out with the potentiometry-based Electronic tongue and the data was analysed using Principle Component Analysis, Discriminant Function Analysis, Taste Discrimination Analysis and Soft Independent Modeling of Class Analogy.

**Results:** Chemosensory similarities were observed between taste standards and the plant samples–citric acid with sour group plants, sweet category plants with sucrose, glycine, β-glucose and D-galactose. The multivariate analyses could discriminate the sweet and sour, sweet and bitter, sweet and pungent, sour and pungent plant groups. Chemosensory category of plant (classified as unknown) could also be identified.

**Conclusion:** This preliminary study has indicated the possibility of fingerprinting the chemosensory-based ayurvedic classification of medicinal plants using E-tongue coupled with multivariate statistical analysis.

## 1 Introduction

The relationship between humans and plants is as old as the history of mankind itself. Infact, plants have been used as medicine since antiquity and continues to be utilised so (Austin, 2003). The early humans must have selected the usable plants and/or plant parts by trial, error, observations (eg., feeding behaviour of animals), empirical reasoning and even some amount of experimentation (Schmidt et al., 2007; Pan et al., 2014; Baluska et al., 2020). Over the millennia, the best among the medicinal plants became part of the tradition of ethnomedicine (Fuller et al., 2019; Akash et al., 2020). In countries like India and China, this information was systematically recorded and incorporated into their indigenous Materia Medica (Mootooswamy, 1886; Touwaide and Appetiti, 2013).

Ayurveda, the medical system indigenous to the Indian subcontinent, is perhaps the longest unbroken health tradition in the world with textual and theoretical backing for its clinical practices (Jayasundar, 2010; Jayasundar, 2017). It has a long history of using medicinal plants for therapeutic and nutritional purposes (Gogte, 2001; Sastry, 2008). To understand medicinal plants and put them to therapeutic use, ayurveda has its own pharmacological metrics (Gogte, 2001; Sastry, 2008). These are articulated and elaborated in ayurvedic pharmacology known as *Dravyaguna Vijñāna*. One such pharmacological metric is *rasa*, which is identified as a chemosensory property. Ayurveda has categorised plants under six *rasas*/tastes: sweet (*madhura*), sour (*amla*), saline (*lavana*), pungent/hot (*katu*), bitter (*tikta*) and astringent (*kashāyā*). Each plant/plant part has one or a combination of *rasas*. For example—*Leucos cephalotus* has a single *rasa* (pungent); *Hydnocarpus wightiana* is pungent and bitter (2 tastes); *Syzygium cumini* is astringent, sweet and sour (3 tastes); *Clerodendrum phlomidis* is bitter, pungent, astringent and sweet (4 tastes); *Terminalia chebula* is astringent, bitter, pungent, sour and sweet (5 tastes) (Gogte, 2001; Sastry, 2008).

Together with other ayurvedic pharmacological parameters like physicochemical properties and pharmacological potency, *rasa* of plants predicts nearly 80% of the pharmacological action from an ayurvedic stance. These ayurvedic parameters are used till date by ayurvedic physicians to decide the usage of medicinal plants for therapeutics. It will be interesting and pertinent to study *rasa* of medicinal plants from the perspectives of contemporary science. At the same time, it is pointed out that taste has always been discussed in modern chemistry in the specific context of food and beverages. However, new paradigms in taste research have emerged, where correlation of taste with pharmacological activities have also been reported (Beauchamp et al., 2005; Kakeda et al., 2010).

Objective evaluation of taste using instruments like Electronic tongue (E-tongue) for industrial applications (pharmaceutical, beverage and tea/coffee industries) has also attracted much attention (Woertz et al., 2011; Latha and Lakshmi, 2012; Podrazka et al., 2018; Liu et al., 2020). There are reports of the use of E-tongue in Traditional Chinese Medicine (TCM) (Kataoka, 2008; Li et al., 2016; Lin et al., 2016; Liang et al., 2017). These studies have focused on the bitterness aspect of the TCMs and herbs. Interestingly, Chinese medicine has also a taste-based classification of medicinal materials. However, unlike ayurveda which mentions six tastes, TCM refers to only five tastes (sweet, salty, sour, bitter, and acrid/pungent) consistent with its five element concept (Hsu et al., 1986; Yarong, 1995). The use of E-tongue in TCM has mostly been restricted to bitter taste of its medicines and some herbs. This study has explored the use of E-tongue coupled with multivariate statistical analysis for studying medicinal plants classified on the basis of their *rasa*/chemosensory properties in ayurveda. This is the first report of use of E-tongue to fingerprint ayurvedic *rasa*-based classification of medicinal plants. The major question addressed in this study is ‘Can E-tongue fingerprint the ayurvedic *rasa*/taste-based classification of medicinal plants?’.

## 2 Materials and methods

### 2.1 Samples

Seventy-eight medicinal plants, belonging to five taste categories (Gogte, 2001; Sastry, 2008), were obtained in dry form from the drug manufacturing unit of Kottakal Arya Vaidya Sala, Kottakkal, India. Of these 78 plant samples, 18 (each) were from sweet and bitter categories, 22 from pungent, 16 from astringent and 4 from sour. It is to be noted that very few plants with predominance of sour taste is mentioned in ayurvedic texts. Of these, only some could be procured thus accounting for fewer plants in the sour category. Since no saline taste plants are mentioned in ayurvedic texts, this taste category has not been included in this study despite the presence of a saline specific sensor. The following taste standards were purchased from Sigma Aldrich: sour—citric acid, hydrochloric acid (HCl); bitter—caffeine, quinine; sweet—L-alanine, glycine, β-glucose, sucrose, D-galactose, cellobiose, arabinose, mannose, maltose, lactose and xylose. Tables 1, 2 list the plant parts used and the botanical names of the samples as provided by the supplier. It is pointed out some of the botanical names listed in the Tables are synonyms (indicated by *) and some are unresolved (indicated by **) (www.theplantlist.org).

**TABLE 1:** Medicinal plants under sweet, bitter and pungent taste categories. wp—whole plant; * synonyms; ** unresolved names; *Vernonia anthelmintica* (L.) Willd–synonym of *Baccharoides anthelmintica* (L.) Moench; *Piper chaba* Hunter - synonym of *Piper retrofractum* Vahl. (www.theplantlist.org).

S.No.	Botanical name of plants/part used	S.No	Botanical name of plants/part used
**Sweet group**		**Bitter group (contd.)**	
1	*Abutilon indicum* (L.) Sweet/root	34	*Solanum nigrum* L.*/wp
2	*Aconitum ferox* Wall/root	35	*Swertia chirata* Buch.-Ham.ex Wall**/wp
3	*Benincasa hispida* (Thunb.)/fruit	36	*Vernonia anthelmintica (L.)* Willd.*/root
4	*Borassus flabellifer* L./fruit	**Pungent group**	
5	*Cissus quadrangularis* L./stem	37	*Alpinia galanga* (L.) Willd/rhizome
6	*Cocos nucifera* L./flower	38	*Anacyclus pyrethrum* (L.) Lag./root
7	*Phoenix sylvestris* (L.) Roxb./fruit	39	*Baliospermum solanifolium* (Burm.) Suresh/root
8	*Cassia fistula* L./root bark	40	*Brassica juncea* (L.) Czern/seed
9	*Glycyrrhiza glabra* L./root	41	*Capsicum annum* L./fruit
10	*Musa paradisiaca* L./tuber	42	*Carum carvi* L./fruit
11	*Leptadenia reticulata* (Retz.) Wight & Arn./root	43	*Cassia tora* L.*/root
12	*Plantago ovata* Forssk./seed	44	*Croton tiglium* L./seed
13	*Phaseolus trilobus* Aiton*/wp	45	*Cuminum cyminum* L./fruit
14	*Prunus amygdalus* Stokes**/seed	46	*Erythrina indica* Lam.*/stem bark
15	*Pueraria tuberosa* (Willd.) DC./tuber	47	*Euphorbia neriifolia L*./leaf
16	*Sida cordifolia* L./root	48	*Ferula narthex* Boiss./resin
17	*Tribulus terrestris* L./fruit	49	*Gossypium herbaceum* L./seed
18	*Vitis vinifera* L./fruit	50	*Leucas cephalotes* (Roth) Spreng./wp
**Bitter group**		51	*Mentha *x* piperita* L./leaf
19	*Andrographis paniculata* (Burm.f.) Nees./wp	52	*Piper chaba* Hunter*/root
20	*Aristolochia bracteolata* Lam./leaf	53	*Piper longum* L./fruit
21	*Centella asiatica* (L.) Urb./wp	54	*Piper longum* L./root
22	*Cissampelos pareira* L./root	55	*Piper nigrum* L./fruit
23	*Citrullus colocynthis* (L.) Schrad./root	56	*Plumbago zeylanica* L./root bark
24	*Coccinia grandis* (L.) Voigt/root	57	*Trigonella foenum-graecum* L./seed
25	*Euphorbia thomsoniana* Boiss./root	58	*Zingiber officinale* Roscoe/rhizome
26	*Gymnema sylvestre* (Retz.) R.Br.ex Sm./leaf		
27	*Indigofera tinctoria* L./wp		
28	*Luffa acutangula* (L.) Roxb./fruit		
29	*Momordica charantia* L./wp		
30	*Nyctanthes arbor-tristis* L./leaf		
31	*Picrorhiza kurroa* Royle ex Benth.**/root		
32	*Rauvolfia serpentina* (L.) Benth. ex Kurz/root		
33	*Smilax glabra* Roxb./rhizome		

**TABLE 2:** Medicinal plants under astringent and sour taste categories. * synonyms; ** unresolved names; *Ougeinia dalbergioides* Benth. - synonym for *Desmodium oojeinense* (Roxb.) H. Ohashi; *Salmalia malabarica* (DC.) Schott and Endl. - synonym of *Bombax ceiba* L. (www.theplantlist.org).

S.No.	Botanical name of plants/part used
**Astringent group**	
1	*Acacia nilotica* (L.) Delile/stem bark
2	*Bauhinia purpurea* L./stem bark
3	*Dolichos biflorus* L.*/seed
4	*Ficus benghalensis* L./stem bark
5	*Ficus lacor* Buch.-Ham/stem
6	*Ficus racemosa* L./stem bark
7	*Ficus religiosa* L./stem bark
8	*Gossypium herbaceum* L./root bark
9	*Mangifera indica* L./seed
10	*Ougeinia dalbergioides* Benth.*/stem
11	*Salmalia malabarica* (DC.) Schott and Endl.*/stem bark
12	*Symplocos racemosa* Roxb./stem bark
13	*Terminalia arjuna* (Roxb. ex DC.) Wight and Arn./stem
14	*Terminalia bellirica* (Gaertn.) Roxb./fruit rind
15	*Thespesia populnea* (L.) Sol. ex Correa/stem bark
16	*Woodfordia floribunda* Salisb.**/flower
**Sour group**	
17	*Citrus medica* L./fruit
18	*Garcinia indica* (Thouars) Choisy/fruit
19	*Tamarindus indica* L./seed
20	*Thespesia populnea* (L.) Sol. ex Correa/fruit

### 2.2 E-tongue

The organoleptic property of taste was evaluated with the potentiometry based Astree Electronic tongue (Alpha MOS, France). The instrument measured the potential differences generated by the sample between sensors and the reference electrode. These were integrated and analysed using the in-built company developed algorithm. The sensor array # 5 used in E-tongue had seven sensors and a reference electrode. Of these, three were tuned each to sour, saline and umami tastes, and the other four sensors gave an integrated response. The electrochemical signals from all the sensors were acquired and stored as data matrix for the chemosensory analysis.

### 2.3 Sample preparation

A 10% aqueous solution of plant samples was prepared. Ten gm of coarsely broken plant samples were soaked in 100 ml of distilled water at 25°C (room temperature) for 24 h, cold macerated, filtered and then centrifuged twice at 5,000 rpm for 10 min each at 20°C. The supernatant was filtered through Whatmann Paper No.1 to remove the very fine suspended particles, lyophilized and the powder stored for further studies. The sample preparation by cold maceration followed the method suggested in ayurvedic texts for assessment of *rasa*/taste (Tripathi, 2006). Taste standards were prepared in distilled water with a concentration of 1 mM.

### 2.4 E-tongue measurements

Twenty-five mg of lyophilized plant samples were dissolved in 100 ml of distilled water. The mixture was kept in water bath for 15 min at 35°C. After stirring for 10 min, the solution was filtered using Whatmann filter paper to remove any suspended particles. Beakers alternatively filled with 100 ml of sample and distilled water (for cleaning sensors) were loaded in the 16 autosampler of the E-tongue (Kumar et al., 2021). The acquisition parameters were: 120 s acquisition time; 10 s sensor cleaning time; 5 replicates per sample.

#### 2.4.1 Taste group identification using taste standards

This study was carried out for the sweet, bitter and sour chemosensory groups. The reference taste standards used were citric acid (sour), caffeine and quinine (bitter), L-alanine, glycine, β-glucose, sucrose, D-galactose, cellobiose, arabinose, maltose, mannose, lactose and xylose (sweet) (Shallenberger, 2012). The sensor response data were simultaneously acquired from the plant samples and the corresponding taste standards. For instance, sensor data from sweet chemosensory group of plants were acquired along with the sweet taste standard. The sensor responses were stored in a single data library and used for the analysis. Multivariate analyses such as Discriminant Function Analysis (DFA), Taste Discrimination Analysis (TDA) and Soft Independent Modeling of Class Analogy (SIMCA) were carried out to study the correlation between the taste of reference compound and plants.

#### 2.4.2 Taste-based differentiation of plants

Sensor response of plants from different taste groups were acquired separately and data library prepared for the different categories. Comparison was made between taste groups using DFA and SIMCA analyses. The results presented are for groups which showed differentiation/fingerprinting—sweet and sour, sweet and bitter, sweet and pungent, and sour and pungent.

#### 2.4.3 Taste ranking of plants

This was carried out only for the sour group of plants since the system had a sensor specific for sour taste. HCl was used as a reference. The sensor responses were assessed on a relative intensity scale of 1–10, from the least to the most intense taste perception. The ranking was carried out by the AlphaMOS analysis software.

#### 2.4.4 Concentration of taste associated phytochemicals in plants

Calibration curve was generated using the sweet, bitter and sour taste standards. These were prepared separately in 100 ml of distilled water in the following concentrations–0.1, 0.2, 0.5, 0.8, 1.0, 1.2, 1.5, 2.0, 4.0, 5.0, 8.0, 10.0, 12.0 and 15 mM. Calibration curve was plotted between concentration and the measured sensor response using Partial least square regression analysis. The data was acquired in triplicate for each concentration. The sensor responses from plant samples were projected onto the calibration curve of their respective taste standards and concentration of these taste associated phytochemicals determined in mM/gm dw (dw—dry weight).

#### 2.4.5 Prediction of taste group of unknown plants

Two plant groups with known class identification (sweet and bitter) and a sample marked as unclassified were selected for this study. Sensor response data from all samples (known and unknown) were acquired simultaneously. The class information of the two groups of plants (sweet and bitter) were entered in the data library and the unknown plant (whose class information was known to be sweet) was marked as unclassified. DFA, SIMCA and TDA were used for the prediction of the taste category of the unknown plant.

### 2.5 Data analysis

All the multivariate analyses were carried out using the manufacturer’s inbuilt software, customised for analysing the sensor response data.

#### 2.5.1 Discriminant function analysis

This supervised analysis used the reduced dimensionality of the data from Principle Component Analysis for quantitative chemosensory differentiation and fingerprinting of the sensor response data from the plants. Intragroup similarities and intergroup discrimination were quantified using Euclidean Distance (ED), which is the distance between the centroids of the groups. The following cut-off values were used for grading ED- <5: poor discrimination and good similarity between groups, 5−20: moderate discrimination, >20: good discrimination and poor similarity between groups. These cut-off values and the ones specified for TDA in the next section were selected by the manufacturer’s inbuilt algorithm.

#### 2.5.2 Taste discrimination analysis

In this analysis, organoleptic distance between the sample and the reference taste standard, quantified in organoleptic units (OU) was used as the discrimination index. The sensor response data converted to organoleptic distance was plotted on the y-axis with plant data points on the x-axis. Shaded area in the graph indicated the region of minimum covariance for the reference taste standard. Deviation from this region indicated dissimilarity with the reference standard. The cut-off values of OU were- < 10: poor discrimination and good similarity, 10−50: moderate discrimination, > 50: good discrimination and poor similarity.

#### 2.5.3 Fingerprinting by class analogy

Soft Independent Modeling of Class Analogy (SIMCA) classified samples into groups. For this, a training data set (target/known class) was first created from taste standards or plant groups with known chemosensory properties. This was used to generate a threshold delineating an acceptance area, shown colored in the SIMCA plot. All data points located within this area were identified with the known class with a Confidence Index (CI) of 95%. Data elements located well beyond the threshold were ‘extreme’ data points, denoting a high level of discrimination between them and the target class. Samples from the training data points, which are not within the acceptance area but just beyond it are outliers and these arise from the internal variances of the sensor response within the training data set. Validation score of 50 was the cut off value for this analysis.

## 3 Results

### 3.1 Taste group identification using taste standards

**FIGURE 1:**
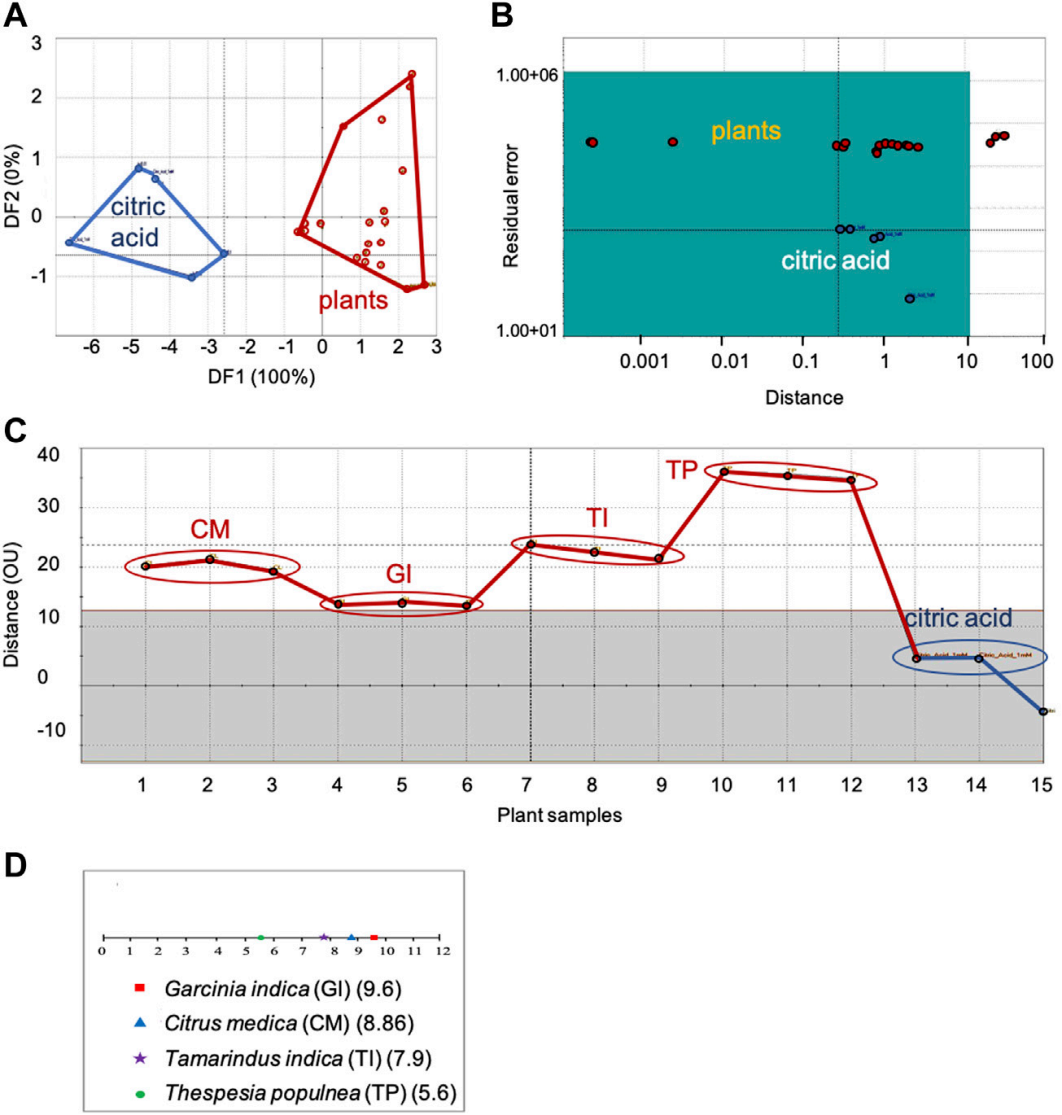
Comparison of sensor response data of sour group of plants with citric acid, the sour taste standard: **(A)** DFA, **(B)** SIMCA, **(C)** TDA, **(D)** Taste ranking. DFA—Discriminant Function Analysis, SIMCA—Soft Independent Modeling of Class Analogy, TDA—Taste Discrimination Analysis.

**FIGURE 2:**
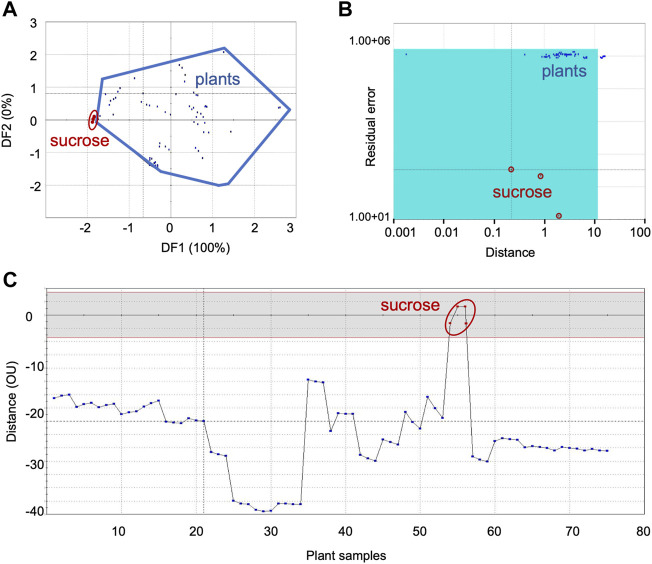
Comparison of sensor response data from sweet group of plants with sucrose, the sweet taste standard: **(A)** DFA, **(B)** SIMCA, **(C)** TDA. DFA—Discriminant Function Analysis, SIMCA—Soft Independent Modeling of Class Analogy, TDA—Taste Discrimination Analysis.

**FIGURE 3:**
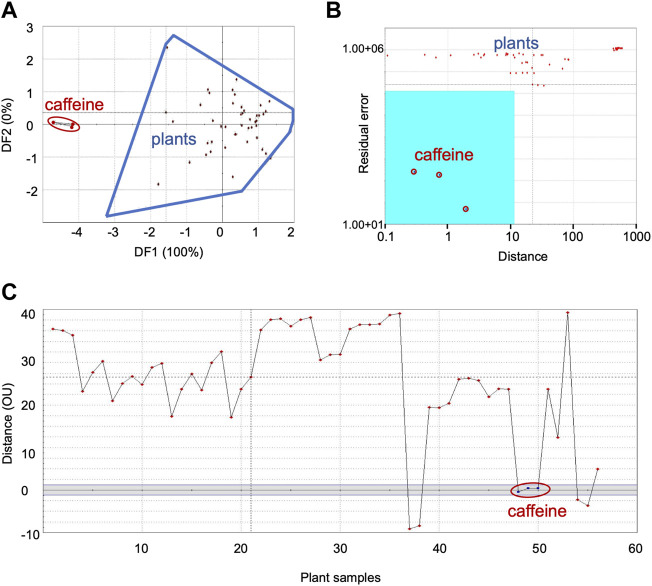
Comparison of sensor response data from bitter group plants with caffeine, the bitter taste standard: **(A)** DFA, **(B)** SIMCA, **(C)** TDA. DFA—Discriminant Function Analysis, SIMCA—Soft Independent Modeling of Class Analogy, TDA—Taste Discrimination Analysis.

Table 3 has summarised the multivariate analyses of the sensor response data of the taste standards and the corresponding chemosensory plant clusters. Sweet and bitter groups were evaluated with more than one taste standard whereas only citric acid was used for the sour group. The chemicals associated with pungency are volatile and insoluble in water. Since the samples for E-tongue measurements should be stable at room temperature and also soluble in water, pungent standards were not used. Pure compounds associated with astringent taste were also not used due to their non-availability. One complete set of data (DFA, SIMCA, TDA) from the sour, sweet and bitter groups are shown as representatives in Figures 1–3. In addition, Figure 1 from sour group of plants has data from taste ranking study as well. The rest of the data is provided as supplementary files (sweet taste group - Supplementary Figures S1–S10; bitter taste group - Supplementary Figure S11).

**TABLE 3:** Results from multivariate analyses of taste standards with plants. AbI- *Abutilon indicum*; AF- *Aconitum ferox*; BC- *Benincasa hispida*; BF- *Borassus flabellifer*; CF- *Cassia fistula*; CM- *Citrus medica*; CN- *Cocos nucifera*; CQ- *Cissus qudrangularis*; GG- *Glycyrrhiza glabra*; GI - *Garcinia indica*; LR- *Leptadenia reticulata*; MP- *Musa paradisiaca*; PA- *Prunus amygdalus*; PO- *Plantago ovata*; PS- *Phoenix sylvestris*; PT- *Phaseolus trilobus*; PuT- *Pueraria tuberosa*; SC- *Sida cordifolia*; TP- *Thespesia populnea*; TT- *Tribulus terrestris*; VV- *Vitis vinifera*; DFA - Discriminant Function Analysis, SIMCA - Soft Independent Modeling of Class Analogy, TDA - Taste Discrimination Analysis; ED - Euclidean Distance; OU - Organoleptic Unit.

Taste standards	Chemosensory association between taste standards and plants using multivariate analysis		
	DFA (ED)	SIMCA	TDA (OU)
**Sour**			
Citric acid	Moderate (6.5)	Good for CM, GI and TP; poor for *Tamarindus indica* L	Moderate (35)
**Sweet**			
L-alanine	Poor (55)	Poor for all plants	Poor (9,000)
Arabinose	Good (1.8)	Poor for all plants	Poor (85)
Cellobiose	Good (1.3)	Poor for all plants	Poor (60)
Dextrose	Moderate (6.5)	Poor for all plants	Poor (400)
D-galactose	Moderate (5)	Good for all plants	Good (4.5)
β-glucose	Good (3.8)	Average for AbI, BF, LR, MP and SC; poor for AF, BC, CF, CN, CQ, GG, PA, PS, PO, PuT, PT, TT and VV	Poor (120)
Glycine	Good (4.3)	Good for all plants	Good (10)
Lactose	Moderate (11.5)	Poor for all plants	Poor (>1,000)
Maltose	Moderate (5)	Poor for all plants	Poor (>1,000)
Mannose	Good (1.5)	Poor for all plants	Poor (>1,000)
Sucrose	Good (1.4)	Good for all plants except CF	Good (4.5)
Xylose	Poor (23.4)	Poor for all plants except CF	Moderate (45)
**Bitter**			
Caffeine	Moderate (11)	Poor for all plants	Good (4)
Quinine	Moderate (7)	Poor for all plants	Moderate (20)

#### 3.1.1 Sour group of plants

Figure 1 shows the DFA (Figure 1A), SIMCA (Figure 1B), TDA (Figure 1C) and taste ranking (Figure 1D) plots for the sour category plants with citric acid as the taste standard.

##### 3.1.1.1 Taste group identification using DFA, SIMCA and TDA

In the DFA plot, clear distinction between the taste standard and the plants was observed along the first axis itself (Figure 1A). The Euclidean Distance was 6.5, indicating moderate similarity between citric acid and the sour category plants. In the SIMCA plot (Figure 1B), all plants except *Tamarindus indica* L. were within the acceptance region of the sour taste standard, indicating good association between citric acid and the sour group of plants. In the TDA analysis, the plants showed moderate similarity (13−35 OU) with the sour taste standard (Figure 1C). Of the sour group plants, *Garcinia indica* (Thouars) Choisy was the closest (least discrimination and maximum similarity with the sensor response to citric acid) with an OU of 13 and *Thespesia populnea* Sol. ex Correa, the farthest with an OU of 35.

##### 3.1.1.2 Taste ranking

Taste ranking of sourness with respect to HCl showed *Garcinia indica* (Thouars) Choisy to be the most sour with a maximum score of 9.6 followed by Citrus medica L. (8.86), *Tamarindus indica* L. (7.9) and *Thespesia populnea* Sol. ex Correa (5.6) (Figure 1D). This ranking order was in complete agreement with the results of TDA, which also showed the chemosensory deviation of plant samples from the taste standard in the same order—maximum sourness for *Garcinia indica* (Thouars) Choisy denoted by its minimum organoleptic distance from the taste standard, and minimum sourness for *Thespesia populnea* Sol. ex Correa, indicated by its maximum OU value.

##### 3.1.1.3 Concentration evaluation

Citrus medica L. exhibited the minimum concentration (2.9 mM/gm dw) and *Thespesia populnea* Sol. ex Correa, the maximum (4.38 mM/gm dw) of citric acid. Both *Tamarindus indica* L. and *Garcinia* indica (Thouars) Choisy showed a concentration value of 3.9 mM/gm dw.

#### 3.1.2 Sweet category

Figure 2 shows the representative data for the sweet category plants with sucrose as the standard—DFA (Figure 2A), SIMCA (Figure 2B) and TDA (Figure 2C). Data for the other sweet taste standards, L-alanine, arabinose, cellobiose, dextrose, D-galactose, β-glucose, glycine, lactose, mannose and xylose are presented respectively, in the Supplementary Figures S1–S10.

##### 3.1.2.1 Taste-based group identification using DFA, SIMCA and TDA

The Euclidean distance between the two groups was 1.4 in DFA, signifying good chemosensory similarities between sucrose and the sweet group of plants (Figure 2A). Like sucrose, significant similarities were also observed in DFA between the sweet group plant samples, and the following taste standards-β-glucose, glycine, mannose, arabinose and cellobiose. At the same time, dextrose, D-galactose, maltose and lactose exhibited moderate similarities with the sweet category plants (Table 3). On the whole, sweet taste associated molecules such as sucrose, glycine, mannose, β-glucose, arabinose, cellobiose, dextrose, D-galactose, maltose and lactose can be considered to have good to moderate chemosensory association with plant samples belonging to the sweet taste group. At the same time, L-alanine and xylose exhibited poor association with this group of plants. All plants except *Cassia fistula* L. (CF) were within the acceptance region of sucrose (validation score 89), showing good chemosensory similarity between the sensor responses of the taste standard and the plants (Figure 2B). Results from TDA showed the organoleptic distance of all plants between 10−45 OU from the standard sucrose (Figure 2C), indicating their moderate level of similarity. In general, sensor response data showed similar trends for SIMCA and TDA analyses for most of the samples.

With glycine and galactose as the reference taste standards, sensor responses from all plants were within the acceptance region of SIMCA, indicating their strong association with these taste standards (Table 3). However, with the taste standard glucose, only five plant samples [*Abutilon indicum* (L.) Sweet, *Borasses flabellifer* L., *Leptadania reticulata* (Retz.) Wight and Arn, *Musa paradisiaca* L., *Sida cordifolia* L.] were in the acceptance region. Other 13 samples were outliers, although their data points were very close to the acceptance region. This can be taken as indicating moderate similarity of these plants with glucose. Arabinose, cellobiose, dextrose and mannose showed organoleptic similarities with the sweet group of plants in DFA but were outliers in SIMCA and TDA plots. None of the analyses showed chemosensory similarities between the plant samples and the taste standard L-alanine.

##### 3.1.2.2 Concentration evaluation

Table 4 lists the concentrations of sweet taste associated phytochemicals in the sweet group of plants. Sucrose registered the highest value (35.1 ± 19.5 mM/gm dw) compared to other sweet taste standards. The very low values of *Aconitum ferox* Wall. and *Cissus quadrangularis* L. have been excluded. *Phoenix sylvestris* (L.) Roxb showed the maximum concentration of sucrose (73.9 mM/gm dw), very closely followed by *Vitis vinifera* L. (72.65 mM/gm dw.) Arabinose had the second highest average concentration (4.93 ± 1.1 mM/gm dw). There were huge variations in the concentrations across the samples, as seen from the SDs. Compounds like arabinose, cellobiose, dextrose, lactose, maltose and mannose, which had the sweet group plants as outliers in SIMCA analysis were in sufficient but low concentration in the plants. It is speculated that the low concentrations of these taste associated molecules in the plants could have led to their reduced sensor response, which in turn could have influenced the multivariate analyses.

**TABLE 4:** Concentration of chemosensory associated phytochemicals from sweet taste category measured by E-tongue. ‘-’ concentration <0.005 mM/gm dw.

S. No	Plants	Concentration (mM/gm dry weight)											
		L-alanine	Arabinose	Cellobiose	Dextrose	D-galactose	β-glucose	Glycine	Lactose	Mannose	Maltose	Sucrose	Xylose
1	*Abutilon indicum*	1.85	5.85	0.34	0.23	2.35	0.53	0.02	−	0.25	−	49	0.02
2	*Aconitum ferox*	0.65	5.13	0.22	0.20	0.01	0.42	-	0.05	0.20	0.01	5.05	−
3	*Benincasa hispida*	1.20	5.88	0.12	0.01	0.21	0.70	0.20	0.06	-	0.01	30.8	−
4	*Borassus flabellifer*	1.37	6.37	0.38	0.37	4.86	0.45	−	0.02	0.37	0.06	37.14	−
5	*Cassia fistula*	0.26	2.82	−	0.03	−	0.20	−	−	0.11	−	42.84	−
6	*Cissus quadrangularis*	0.65	4.55	0.05	0.19	1.13	0.11	0.04	0.11	−	0.04	3.308	−
7	*Cocus nucifera*	0.52	5.42	0.22	−	0.50	0.37	−	0.22	−	−	32.96	0.11
8	*Glycyrrhiza glabra*	0.31	5.31	0.01	0.31	−	0.37	5.28	0.20	−	−	35.31	0.03
9	*Leptadenia reticulata*	0.96	5.96	0.12	0.35	2.03	0.50	−	−	0.36	0.36	37.75	−
10	*Musa paradisiaca*	0.96	5.96	0.09	0.55	0.01	0.21	12.9	0.21	−	−	50.24	−
11	*Phaseolus trilobus*	0.25	3.1	−	−	2.20	0.02	0.51	0.51	0.06	−	11.73	0.05
12	*Phoenix sylvestris*	0.96	4.97	−	0.36	−	0.62	11.93	−	0.14	0.04	73.9	−
13	*Plantago ovata*	0.59	3.8	0.2	−	−	0.42	−	−	0.11	0.21	40.8	−
14	*Prunus amygdalus*	0.21	3.91	−	−	0.88	0.08	−	−	−	0.01	14.8	0.02
15	*Pueraria tuberosa*	0.50	3.84	0.01	−	−	0.21	−	0.05	−	−	24.4	−
16	*Sida cordifolia*	0.53	5.33	0.23	0.20	3.24	0.23	−	−	0.43	0.43	37.75	−
17	*Tribulus terrestris*	0.31	4.21	0.13	−	0.64	0.32	1.24	0.40	0.02	−	30.85	0.02
18	*Vitis vinifera*	0.25	6.4	0.09	0.22	0.01	0.28	6.32	0.01	−	−	72.65	0.32
	Mean + SD	0.69 + 0.45	4.93 + 1.10	0.16 + 0.11	0.25 + 0.15	1.39 + 1.48	0.34 + 0.19	4.28 + 5.17	0.17 + 0.16	0.21 + 0.14	0.13 + 0.16	35.07 + 19.52	0.08 + 0.11

#### 3.1.3 Bitter category

Data with caffeine as the reference taste standard is shown as representative of bitter category in Figure 3—plots of DFA (Figure 3A), SIMCA (Figure 3B) and TDA (Figure 3C). Supplementary Figure S11 shows the data for quinine.

##### 3.1.3.1 Taste group identification using DFA, SIMCA and TDA

DFA showed distinct data clusters for the taste standard and the plant data but an ED of 8 indicated only moderate similarity between them (Figure 3A). Quinine also showed moderate similarity with plant samples in DFA analysis (Table 3). SIMCA analysis showed poor association between the taste standard and the bitter group plants, with sensor response data from all the plants plotting outside the acceptance region (validation score-89) (Figure 3B). The TDA plot showed all data points from the plants outside the grey region (Figure 3C). The organoleptic distance was > 10 OU and indicated moderate organoleptic association between the taste standard and the bitter group of plants.

##### 3.1.3.2 Concentration evaluation

Table 5 lists the concentrations of caffeine and quinine in the plants. *Swertia chirata* Buch.-Ham.ex Wall showed the highest concentration (4.32 mM/gm dw) for caffeine and *Cissampelos pareira* L., the maximum (0.51 mM/gm dw) for quinine. The concentrations of bitter taste associated molecules were generally lower than those observed in the sweet and sour group of plants.

**TABLE 5:** Concentration of chemosensory associated phytochemicals from bitter taste category measured by E-tongue. ‘-’ concentration <0.001 mM/gm dw.

S. No	Plants	Concentration (mM/gm dry weight)	
		Caffeine	Quinine
1	*Andrographis paniculata*	2.06	0.32
2	*Aristlochia bracteolate*	−	−
3	*Centella asiatica*	0.25	−
4	*Cissampelos pariera*	−	0.51
5	*Citrullus colocynthis*	2.10	0.11
6	*Coccinia grandis*	0.21	−
7	*Euphorbia thomsoniana*	−	0.20
8	*Gymnema sylvestre*	0.51	−
9	*Indigofera tinctoria*	2.2	0.22
10	*Luffa acutangula*	1.4	−
11	*Momordica charantia*	4.24	0.20
12	*Nyctanthes arbor-tristis*	0.04	−
13	*Picrorhiza kurroa*	−	0.31
14	*Rauvolfia serpentina*	0.79	0.21
15	*Smilax glabra*	−	−
16	*Solanum nigrum*	0.25	−
17	*Swertia chirata*	4.32	0.32
18	*Vernonia anthelmintica*	1.60	0.01
	Mean ± SD	1.54 ± 1.44	0.24 ± 0.14

### 3.2 Taste-based differentiation of plants

**FIGURE 4:**
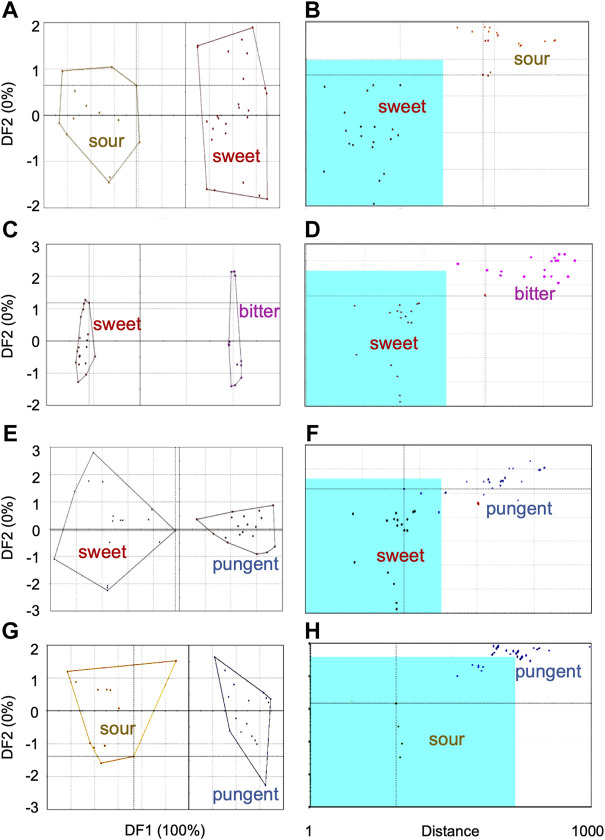
Chemosensory-based discrimination of sensor response data from plant groups using multivariate analyses—**(A,C,E,G)** Discriminant Function Analysis, **(B,D,F,H)** Soft Independent Modeling of Class Analogy. Comparison between sensor responses from chemosensory groups are shown—**(A,B)** sweet and sour, **(C,D)** sweet and bitter, **(E,F)** sweet and pungent, **(G,H)** sour and pungent.

DFA of sensor response data from all samples pooled together exhibited discrimination between sweet category and sour, bitter, astringent and pungent groups in the DF2 axis only. For clearer differentiation, sensor data was compared in sets of two chemosensory groups. Results from those which showed discrimination are presented in Figure 4 [DFA (Figures 4A,C,E,G) and SIMCA (Figures 4B,D,F,H)]. TDA analysis of the sensor response data from the five taste categories also showed chemosensory-based differentiation between the plant samples. Minimum variability was observed within sweet group (OU < 10) and good separation with moderate similarity between sweet and other groups, namely sweet and sour (35 OU); sweet and bitter (17 OU), sweet and pungent (20 OU), and sweet and astringent (18 OU).

#### 3.2.1 Sweet with other taste categories

##### 3.2.1.1 Sweet and sour

In the discriminant analysis, the two taste categories showed moderate discrimination with an ED of 6.2 (Figure 4A). In the SIMCA plot (Figure 4B), all plants from sour category plotted well outside the acceptance region of sensor data from the sweet group of plants. This confirmed that sensor responses of plants from sour group were significantly different from those of sweet (Figure 4B). At the same time, two plants [*Phoenix sylvestris* (L.) Roxb. and *Glycyrrhiza glabra* L.] from the training data set of sweet category were outliers, indicating their internal data variations.

##### 3.2.1.2 Sweet and bitter

In DFA (Figure 4C), DF1 showed good discrimination between sweet and bitter groups with an ED of 30. In the SIMCA analysis with sweet group as the trained data set (validation score–100), sensor data from all bitter category plants plotted outside the acceptance region (Figure 4D), indicating good discrimination between the two chemosensory groups. *Phoenix sylvestris* (L.) Roxb. was an outlier for the training data set. The taste discrimination analysis between the two taste groups also demonstrated good discrimination with a maximum OU of 40.

##### 3.2.1.3 Sweet and pungent

DFA discriminated moderately (ED = 5.5) between the sensor responses of the sweet and pungent group of plants (Figure 4E). In the SIMCA plot (Figure 4F), all pungent category plants (except *Cuminum cyminum* L.) plotted outside the acceptance region, containing sensor response data from plants in sweet category (Figure 4F). This indicated good chemosensory discrimination between the plants from the two taste groups. Taste discrimination analysis demonstrated moderate discrimination with a maximum OU of 45 between the sweet and pungent group of plants.

### 3.2.2 Sour and pungent categories

DFA showed moderate discrimination (ED = 5.8) between the sour and pungent category (Figure 4G). All plants from the pungent group (except two) plotted outside the acceptance region (sour group) of the SIMCA plot (Figure 4H), signifying discrimination between the two chemosensory categories. However, since the validation score of 45 for this model was less than the cut off, results from SIMCA were not considered significant for these two category of plants.

### 3.3 Predicting taste of unknown plants

**FIGURE 5:**
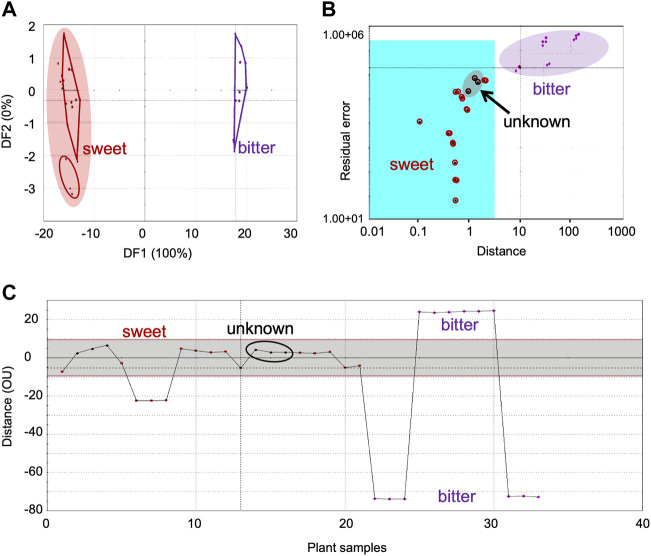
Multivariate analyses to identify the chemosensory group of unknown sample: **(A)** DFA, **(B)** SIMCA, **(C)** TDA. DFA—Discriminant Function Analysis, SIMCA—Soft Independent Modeling of Class Analogy, TDA—Taste Discrimination Analysis.

In DFA, the discrimination index between the sensor response from the unknown and bitter group plants was 17.8 (ED) (Figure 5A), indicating moderate discrimination. This ED value was nearer to the upper limit of the ‘moderate discrimination’ window. On the other hand, ED between the unknown and sweet category was 0.38, confirming good similarity between them. Results from SIMCA (validation score–82) also showed the unknown plant (shown highlighted) within the acceptance region of sweet category indicating the class identification for this plant as sweet taste (Figure 5B). In TDA also, the sensor response from the unknown plant grouped with the sweet category (OD < 10) and differed from the bitter category (OD > 10) (Figure 5C). These confirmed the (already known) class of this plant to be sweet.

## 4 Discussion

Despite the success of synthetic drugs in modern medicine over the past century, statistics indicate that plant-based remedies meet the healthcare needs of nearly 80% of the world population, especially in rural areas (Bodeker et al., 2005; Burke et al., 2022). At the same time, there is also a growing interest world over in the use of traditional medical systems, which are vast repositories of plant-based medicines. This brings into context ayurveda, which has a long tradition of use of medicinal plants. Ayurvedic pharmacology has its own metrics such as *rasa* (taste) for understanding the therapeutic properties of medicinal plants.

All medicinal plants used in ayurveda are classified according to the six *rasas* (sweet, sour, saline, pungent, bitter, astringent). From an ayurvedic stance, plants grouped under one taste category have commonalities, enabling their use for specific clinical conditions. However, from the perspective of modern botany, there are no pharmacological similarities between the plants grouped under one taste category. For example, the taxonomic nomenclature for *Abutilon indicum* from the sweet group (Table 1) is as follows: Order—Malvales, Family—Malvaceae; Genus—Abutilon Mill.; Species—Abutilon indicum (L.) Sweet (https://www.itis.gov/servlet/SingleRpt/SingleRptsearch_topic=TSN&search_value=21682#null). It has alkaloids, saponins, flavonoids, glycosides, and some essential oils like asparagines and geraniol (Venkat and Suvarna, 2020). On the other hand, the taxonomic hierarchy of *Vitis vinifera*, also from the sweet group (Table 1) is different: Order—Vitales, Family—Vitaceae; Genus—Vitis L.; Species—Vitis vinifera L. (https://www.itis.gov/servlet/SingleRpt/SingleRptsearch_topic=TSN&search_value=28629#null). The chemical constituents of Vitis vinifera are mainly sugars, phenolic compounds, aromatic acids, flavonoids, proanthocyanidins, and stilbenoids (Insanu et al., 2021) and are different from those of *Abutilon indicum*. These two plants are grouped under the same chemosensory category in ayurveda but will not be under the same category from a modern botany point of view because of the differences in their nomenclature and phytochemical composition.

It is against this background that this exploratory work has been planned, conducted and articulated. In an effort to address the ayurvedic *rasa*-based classification of medicinal plants, the technique of E-tongue has been used. Since this technique is generally not used to study whole extracts of plants, there were many challenges and hurdles necessitating extensive control and standardization experiments (Jayasundar et al., 2021; Kumar et al., 2021). The potential of this analytical technique for fingerprinting of taste-based categorization of medicinal plants has been explored for the very first time.

Discussing the results—In the SIMCA analysis of the sour plant category (Figure 1C), *Tamarindus indica* L. was an outlier with respect to citric acid, the sour reference taste standard. Although presence of citric acid in *Citrus medica* L. and *Garcinia indica* (Thouars) Choisy are reported in literature (Jayaprakasha et al., 2002), there are no such reports for *Thespesia populnea* (L.) Sol. ex Correa and seeds (the part used in this study) of *Tamarindus indica* L. As for the results from the sweet group of plants and taste standards—All the sweet taste standards used were primary metabolites (sugars and amino acids) and produced by all plants (Balandrin, 1985; Maeda, 2019). For example, glycine, which showed good association in all analyses, is a non-essential amino acid produced by all plants (Buchanan et al., 2015). L-alanine, another amino acid, is generally produced by plants in very low concentration (Buchanan et al., 2015). Its absence in the various analyses could be due to its inherently low concentration leading to its reduced/absent sensor response.

In taste chemistry, sucrose is considered the best reference standard for sweetness (Shallengerger, 2012). A high level of chemosensory similarity between sucrose and the plants under sweet category was observed in both DFA (ED= 1.4) and SIMCA analyses (Table 3). This indicated that *rasa* could imply taste as understood in modern chemistry. At the same time, glucose an important sugar molecule produced by all plants, showed only moderate similarity with the sweet group plants compared to sucrose. These results, inferences and discrepancies point to the fact that chemosensory property of complex plant matrices requires further in-depth experiments and analyses.

The bitter phytochemical standards used in this study are found only in some plants - caffeine in *Coffea arabica*, *Cola nitida*, *Theobroma cacao* and *Camellia sinensis* (Graham, 1978; Ashihara and Suzuki, 2004) and quinine in *Cinchona officinalis* (Rates, 2001). Since these taste standards are not reported in the bitter group plants evaluated in this study, their poor association with the plant samples is not surprising. In general, there were differences in the results and inferences from the various analyses. The reasons for this may lie in the variances in the sensor response thresholds used for the various analyses, and the differences in the sample matrix of taste standards and plant samples. The latter are a mixture of several phytochemicals whereas standards are single molecules. Since sensor response is modulated by the sample matrix, and different thresholds are also used for different analyses, discrepancies are expected between the various analyses.

Commenting on the differences in the results obtained with SIMCA and concentrations (Tables 3, 4, 5)—in SIMCA analysis, threshold values and internal variabilities affect the final results. For example, data points below the threshold value will be absent in the acceptance region and those with large internal variance will be outliers. On the other hand, sensor responses from plant samples are directly projected onto the calibration curve without any thresholding for concentration estimation. Moreover, there is also the basic difference in the sample matrix of the taste standards (single molecules) and plant samples (complex mixtures). In addition, the concentrations could not be cross-checked with phytochemical analysis or other advanced quantitative techniques like HPLC, LCMS, etc. Therefore, the concentrations presented should be considered only as indicators of the trend rather than as absolute values. It is pointed out that E-tongue is not the ideal technique to measure the concentrations of phytochemicals. Since E-tongue is designed to study complex matrices, the calibration method was used to study trends in concentrations in the plant samples. However, several in-depth control experiments were carried out prior these studies. In this study, similar trends were observed for SIMCA and TDA in most of the samples. SIMCA used training data set of taste standards and/or plant samples whereas TDA used only taste standards.

‘*Rasa*’, the ayurvedic pharmacological parameter is clearly mentioned in ayurvedic text as the ‘gustatory effect of contact of a substance (dry or wet) with the tongue’ (Sharma and Dash, 2008). According to modern chemistry, taste is a chemosensory property reflecting both the constituent chemicals/molecules and their sensory nature. A relook at the old concept of *rasa* has therefore been attempted from the contemporary perspective of molecules as well. The analytical technique of E-tongue has been used for this purpose to study the relationship between taste associated molecules and plant samples. The main objective of this study however, is to probe if E-tongue can fingerprint the ayurvedic *rasa*/taste-based classification of medicinal plants. This E-tongue based exploratory and preliminary study has indicated that fingerprinting of the chemosensory nature of plants is possible. Further in-depth studies and analyses are underway.

The questions posed in this study have tremendous practical implications for ayurvedic science. Evaluation of *rasa* of plants is a fundamental requirement of ayurvedic pharmacology. Currently, the information on *rasa* of medicinal plants documented in the ayurvedic texts are used. The results of this study indicate that E-tongue has the potential to identify the *rasa* category of known plants and classify those of unknown plants from an ayurvedic perspective. Use of instruments and analytical methodologies provide scientific means for identifying the *rasa* of plants. The technique can also be used as quality control for medicinal plants and identifying adulterants from an ayurvedic standpoint. The study can serve as a starting point for assessing the *rasa* of plants in countries other than India, paving the way for them to make use of their own flora and fauna to prepare ayurvedic medicines.

At the same time, chemosensory property of medicinal plants is a completely novel parameter for plant scientists/pharmacologists and hence could appeal to their scientific curiosity, especially since there are increasing number of reports on the roles of taste in pharmacology and diseases like obesity (Beauchamp et al., 2005; Huang et al., 2017; Liu et al., 2018; Mameli et al., 2019). There are vast repository of plants worldwide waiting to be therapeutically explored. This study would help in identification of therapeutically important unknown plants based on the knowledge of *rasa* identification and could lead to new drug discovery. The present work, first of its kind, is hence important and a step towards evaluating and understanding the *rasa*-based ayurvedic classification of medicinal plants. The study has not only opened new applications for E-tongue in the field of medicinal plants but has also initiated the process of validation of the ayurvedic classification of medicinal plants.

## Data Availability

The original contributions presented in the study are included in the article/Supplementary Material. Further inquiries can be directed to the corresponding author.

## References

[B1] Akash A. Navneet N. Bhandari B. S. (2020). Ethnomedicinal plant use and practice in traditional medicine. Pennysylvania IGI Glob. 10.4018/978-1-7998-1320-0

[B46] Ashihara H. Suzuki T. (2004). Distribution and biosynthesis of caffeine in plants. Front Biosci. 9, 1864–1876. 10.2741/1367 14977593

[B2] Austin D. F. (2003). Plants for people. Econ. Bot. 57, 668. 10.1663/0013-0001 DFABRE]2.0.CO;2

[B3] Balandrin M. F. Klocke J. A. Wurtele E. S. Bollinger W. H. (1985). Natural plant chemicals: Sources of industrial and medicinal materials. Science 228, 1154–1160. 10.1126/science.3890182 3890182

[B4] Baluška F. Mancuso S. (2020). Plants, climate and humans: Plant intelligence changes everything. EMBO Rep. 21, e50109. 10.15252/embr.202050109 e50109 32103598 PMC7054678

[B5] Beauchamp G. K. Keast R. S. J. Morel D. Lin J. Pika J. Han Q. (2005). Phytochemistry: Ibuprofen-like activity in extra-virgin olive oil. Nature 437, 45–46. 10.1038/437045a 16136122

[B6] Bodeker C. Bodeker G. Ong C. K. Grundy C. K. Burford G. Shein K. (2005). WHO global atlas of traditional complementary and alternative medicine. Geneva: World Health Organization.

[B7] Buchanan B. B. Gruissem W. Jones R. L. (2015). Biochemistry and molecular biology of plants. New Jersey, NJ, USA: Wiley Blackwell.

[B8] Burke R. Sherwood O. L. Clune S. Carrollo R. McCabe P. F. Kane A. (2022). Botanical boom: A new opportunity to promote the public appreciation of botany. Plants People Planet 4, 326–334. 10.1002/ppp3.10257

[B9] Fuller D. Q. Murphy C. Kingwell-Banham E. Castillo C. C. Naik S. (2019). Cajanus cajan (L.) Millsp. origins and domestication: The south and southeast asian archaeobotanical evidence. Genet. Resour. Crop Evol. 66, 1175–1188. 10.1007/s10722-019-00774-w

[B10] Gogte V. M. (2001). Ayurvedic pharmacology and therapeutic uses of medicinal plants. Varanasi: Chaukhambha Sanskrit Series Office.

[B11] Graham D. M. (1978). Caffeine—Its identity, dietary sources, intake and biological effects. Nutr. Rev. 36, 97–102. 10.1111/j.1753-4887.1978.tb03717.x 353595

[B12] Hsu H. Y. Chen Y. P. Jyl S. (1986). Oriental Materia medica: A concise guide. Long Beach CA: Oriental Healing Arts Institute. 13:978-0941942225.

[B13] Huang Z. Huang S. Cong H. Li Z. Li J. Keller K. L. (2017). Smell and taste dysfunction is associated with higher serum total cholesterol concentrations in Chinese adults. J. Nutr. 147, 1546–1551. 10.3945/jn.117.250480 28615376 PMC5525109

[B15] Insanu M. Karimah H. Pramastya H. Fidrianny I. (2021). Phytochemical compounds and pharmacological activities of Vitis vinifera L.: An updated review. Biointerface Res. Appl. Chem. 11, 13829. 10.33263/BRIAC115.1382913849

[B16] Jayaprakasha G. K. Sakariah K. K. (2002). Determination of organic acids in leaves and rinds of Garcinia indica (Desr.) by LC. J. Pharm. Biomed. Anal. 28, 379–384. 10.1016/s0731-7085(01)00623-9 11929682

[B17] Jayasundar R. (2010). Ayurveda: A distinctive approach to health and disease. Curr. Sci. 98, 908–914.

[B18] Jayasundar R. (2017). Ayurveda: If systems approach is the way forward, what can the theory of tridosha teach us? Curr. Sci. 112, 1127–1133.

[B19] Jayasundar R. Singh A. Kumar D. (2021). Challenges in using electronic tongue to study rasa of plants: I. Finding the right tool for the right job. J. Ayu. Integr. Med. 12, 234–237. 10.1016/j.jaim.2020.12.011 PMC8185964 33514460

[B20] Kakeda T. Ogino Y. Moriya F. Saito S. (2010). Sweet taste-induced analgesia: An fMRI study. Neuroreport 21, 427–431. 10.1097/WNR.0b013e3283383df5 20220542

[B21] Kataoka M. Tokuyama E. Miyanaga Y. Uchida T. (2008). The taste sensory evaluation of medicinal plants and Chinese medicines. Intl J. Pharm. 351, 36–44. 10.1016/j.ijpharm.2007.09.017 17976934

[B22] Kumar D. Singh A. Jayasundar R. (2021). Challenges in using electronic tongue to study rasa of plants: II. Impact of solvent and concentration on sensor response and taste ranking. J. Ayu. Integr. Med. 12, 238–244. 10.1016/j.jaim.2020.12.010 PMC8185975 33551338

[B23] Latha R. S. Lakshmi P. K. (2012). Electronic tongue: An analytical gustatory tool. J. Adv. Pharm. Technol. Res. 3, 3–8. 10.4103/2231-4040.93556 22470887 PMC3312724

[B24] Li X. Gao X. Liu R. Wang J. Wu Z. Zhang L. (2016). Optimization and validation of the protocol used to analyze the taste of traditional Chinese medicines using an electronic tongue. Exptl Ther. Med. 12, 2949–2957. 10.3892/etm.2016.3733 27882100 PMC5103729

[B25] Liang X. Li B. Wu F. Li T. Wang Y. Ma Q. (2017). Bitterness and antibacterial activities of constituents from Evodia rutaecarpa. BMC Comp. Alt. Med. 17, 180. 10.1186/s12906-017-1701-8 PMC5372309 28356098

[B26] Lin Z. Zhang Q. Liu R. Gao X. Zhang L. Kang B. (2016). Evaluation of the bitterness of traditional Chinese medicines using an E-tongue coupled with a robust partial least squares regression method. Sensors 16, 151. 10.3390/s16020151 26821026 PMC4801529

[B27] Liu J. Zuo M. Low S. S. Xu N. Chen Z. Lv C. (2020). Fuzzy evaluation output of taste information for liquor using electronic tongue based on cloud model. Sensors 20, 686. 10.3390/s20030686 32012652 PMC7038490

[B28] Liu Y. H. Huang Z. Vaidya A. Li J. Curhan G. C. Wu S. (2018). Longitudinal study of altered taste and smell perception and change in blood pressure. Nutr. Metab. Cardiovasc. Dis. 28, 877–883. 10.1016/j.numecd.2018.05.002 29858155 PMC6428580

[B29] Maeda H. A. (2019). Evolutionary diversification of primary metabolism and its contribution to plant chemical diversity. Front. Plant Sci. 10, 881. 10.3389/fpls.2019.00881 31354760 PMC6635470

[B30] Mameli C. Cattaneo C. Panelli S. Comandatore F. Sangiorgio A. Bedogni G. (2019). Taste perception and oral microbiota are associated with obesity in children and adolescents. PLoS One 14, e0221656. 10.1371/journal.pone.0221656 31509575 PMC6738620

[B31] Mootooswamy P. S. (1886). Contributions to the Indian Materia medica. Indian Med. Gazette. 90, 325–328. PMC5000929 28999529

[B32] Pan S. Y. Litscher G. Gao S. H. (2014). Historical perspective of Traditional indigenous medical practices: The current renaissance and conservation of herbal resources. Evid. Based Compl. Alt. Med., 525340. 10.1155/2014/525340 PMC4020364 24872833

[B33] Podrazka M. Baczynska E. Kundys M. Jelen P. S. Nery E. W. (2018). Electronic tongue-A tool for all tastes? Biosensors 8, 3. 10.3390/bios8010003 PMC5872051 29301230

[B34] Rates S. M. K. (2001). Plants as source of drugs. Toxicon 39, 603–613. 10.1016/S0041-0101(00)00154-9 11072038

[B35] Sastry J. L. N. (2008). Dravyaguna Vijnana. Varanasi: Chaukhamba Orientalia.

[B36] Schmidt B. M. Ribnicky D. M. Lipsky P. E. Raskin I. (2007). Revisting the ancient concept of Botanical therapeutics. Nat. Chem. Biol. 3, 360–366. 10.1038/nchembio0707-360 17576417

[B37] Shallenberger R. S. (2012). Taste chemistry. Dordrecht: Springer Science & Business Media.

[B38] Sharma R. K. Dash B. Caraka S. (2008). Chowkhamba Sanskrit series, 1. Varanasi, 459.

[B39] Touwaide A. Appetiti E. (2013). Knowledge of eastern Materia medica (Indian and Chinese) in pre-modern mediterranean medical traditions. A study in comparative historical Ethnopharmacology. J. Ethnopharmacol. 148, 361–378. 10.1016/.j.jep.2013.03.068 23567031

[B40] Tripathi B. (2006). Sharangdhar Samhita. Varanasi: Chaukhamba Surbharti Prakashan, 429–431.

[B41] Venkat S. S. Suvarne R. U. (2020). A review on phytochemical constituents of Abutilon indicum (link) sweet – An important medicinal plant in ayurveda. Plantae Sci. 3, 15–19. 10.32439/ps.v3i3.15-19

[B43] Woertz K. Tissen C. Kleinebudde P. Breitkreutz J. (2011). Taste sensing systems (electronic tongues) for pharmaceutical applications. Intl. J. Pharm. 417, 256–271. 10.1016/j.ijpharm.2010.11.028 21094230

[B44] Yarong H. (1995). Advanced textbook of Traditional Chinese Medicine and pharmacology. State Administration of Traditional Chinese Medicine. Volume II. Beijing, China: New World Press.

